# Our experience in two cases of type IV laryngotracheoesophageal cleft (LTEC) with a diagnosis of antenatal esophageal atresia

**DOI:** 10.11604/pamj.2017.26.55.10647

**Published:** 2017-02-01

**Authors:** Kaan Sonmez, Ramazan Karabulut, Zafer Turkyilmaz, Canan Turkyilmaz, Berrin Isik, Sibel Eryilmaz, Kıvanc Seref, Ebru Ozcan, Gul Meral Hosgoren, Abdullah Can Basaklar

**Affiliations:** 1 Gazi University, Faculty of Medicine, Departments of Pediatric Surgery, Ankara, Turkey; 2 Neonatology Gazi University, Faculty of Medicine, Ankara, Turkey; 3 Anesthesiology and Reanimation, Gazi University, Faculty of Medicine, Ankara, Turkey

**Keywords:** Type IV laryngotracheoesophageal cleft, treatment, anterior cervicothoracic approach

## Abstract

Laryngotracheoesophageal clefts (LTECs) are rare congenital defects that are often accompanied by additional anomalies. The major issues in the treatment of these patients are intraoperative exposure insufficiency, technical difficulty of the operation, and anesthesia problems originating from the respiratory tract. Problems originating from mechanical ventilation and respiratory tract, eating disorders and relapse of fistula are among the problems encountered following surgery. Most of the time, concomitant additional anomalies also worsen the clinical picture. It was our aim with these case reports to report our experience in two cases with Type IV LTEC ranging from the inoperable type IV LTEC due to additional anomalies mounted up to severe respiratory distress to the carina that we operated on with a single stage anterior cervicothoracic approach on its fifth day on life.

## Introduction

Laryngotracheoesophageal cleft (LTEC) is a rare disorder that is often accompanied by serious additional anomalies. While congenital laryngeal anomalies are seen one in every 2000 live births, less than 0.3% of these are LTECs [1]. Laryngeal clefts are classified as type 1: supraglottic interarytenoid cleft; type II: partial cricoid cleft extending below the level of the vocal folds; type III: total cricoid cleft that may extend to the cervical tracheoesophageal septum; and type IV: laryngoesophageal cleft involving a major part of the tracheoesophageal wall in the thorax [2]. Out of these, types III and IV are the most challenging to diagnose and manage since they are life-threatening conditions [1, 2]. It was our aim with these case reports to report our clinical experience in two cases diagnosed with Type IV LTEC.

## Patient and observation

### Case 1

Baby born 3100 gr with a C/S and an APGAR score of 3 and 5 in minutes 1 and 5 respectively and diagnosed with esophagus atresia was intubated due to advancing respiratory distress and was put on mechanical ventilation. The endotracheal tube was often obstructed and pulmonary sounds could be heard from the stomach. When nasogastric tube was inserted by us, it was seen that it was inserted into the stomach, and the diagnosis of esophagus atresia was withdrawn. However, it was seen during intubation that LTEC-concordant trachea and esophagus differentiation was not made. Computed tomography confirmed the diagnosis of type IV LTEC extending to 1.5 cm above of the carina, and it was observed following this level that the trachea and major bronchi were 2 mm and the esophageal lumen was larger. The infant could not be operated on due to concomitant additional anomalies (VACTERL association) and serious pulmonary problems, and was lost on day five.

### Case 2

Baby boy born 2800 gr with a C/S in the 36th week of pregnancy and with an APGAR score of 7 and 8 in minutes 1 and 5 respectively developed respiratory distress and put on mechanical ventilation. Following this, it was seen that the nasogastric tube could not be pushed into the stomach and could not go further than 12 cm, and upon seeing the stomach on abdominal graphy, proximal esophagus atresia with distal fistula was considered. The patient had VACTERL association. On the second day, classic right thoracotomy and extrapleural esophagus atresia repair was attempted. However, it was understood that the patient had lacked upper esophagus and the esophagus had progressed as single lumen while observing that the distal esophagus had penetrated into the tracheal bifurcation widely. Thoracotomy was closed and bronchoscopy was performed on the patient who was diagnosed with type IV LTEC, and the procedure was discontinued for further examination and investigation of the patient and team preparation. Had the anomaly at the entry points of the trachea and esophagus been detected during the first intubation, the first thoracotomy might not have been necessary. Computed tomography confirmed the diagnosis of type IV LTEC extending to the right main stem bronchus (level of carina). After having made all the necessary preparations, the patient was operated on by the anterior cervicothoracic approach described by Lipshutz et al. on day 5 [3]. The infant was positioned with the neck maximally extended, and low transverse neck incision with a lower midline “T” extension and upper median sternotomy through the 1.5 cm manubrium was done. However, since it was not possible to go down to the level of the carina with the described method, the sternum was cut 2 cm more. The trachea was divided along the anterior midline from the larynx to the carina. Meanwhile, it was seen that the esophagus started from the level of the carina close to the right major bronchus.

**Figure 1:**
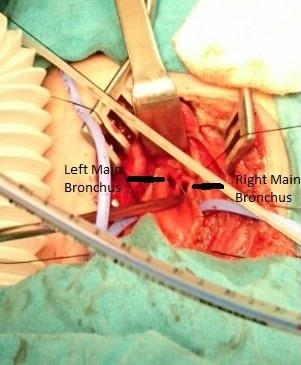
Right and left main bronchi are seen associated with LTEC

**Figure 2:**
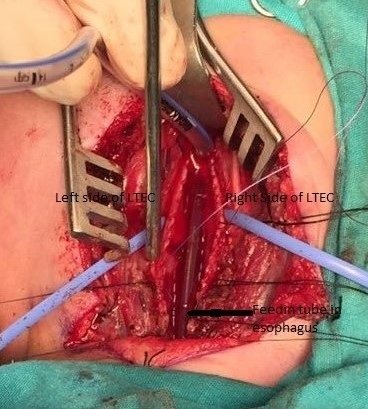
The esophagus was catheterised with a 6 F feeding tube during operation

However, it was so narrow to let the 6f feeding tube to be inserted, and it was pushed to the stomach with difficulty (Figure 1, Figure 2). At this stage, the patient was tried to be respired by intubating the right and left major bronchi intraoperatively and having the right and left bronchi to respire separately. Left intubation was especially more effective. In the meantime, the integrity of the esophagus was ensured with a 5/0 polyglcatin by cutting the esophagus from the right and left posterior wall over the feeding tube. Following this, the two-piece trachea was repaired on the posterior and anterior walls respectively, with the same suture in the manner that it could fit into a 2f endotracheal tube. The patient went into cardiac arrest and was resuscitated twice on the operation table. A chest tube was inserted for right pneumothorax in the thoracal X-ray graphy taken intraoperatively. After having completed the separation of the trachea and esophagus of the patient, anterior cervicothoracic incision was closed and a feeding jejunostomy was inserted by surgically. The patient was followed by having him put on mechanical ventilation. The patient’s hypoxic status continued, his renal functions deteriorated, and despite all mechanic ventilation and cardioprotective support, the patient had cardiac arrest on the second day and did not respond to resuscitation.

## Discussion

Laryngotracheoesophageal clefts (LTEC) are rare but have a high mortality rate. Moreover, just as the endotracheal tube can migrate into the esophagus, the nasogastric the can also translocate towards the trachea. Definitive diagnosis is made during endoscopy, but may be suggested during an esophageal contrast study. Endoscopically, the cleft can easily be missed since it is narrow, and the edges tend to co-apt from the pressure of the tracheal rings. Once diagnosed, definitive surgical repair should be quickly undertaken as life-threatening pulmonary complications may develop, especially with long clefts [4, 5]. LTEC may imitate the clinical picture and diagnosis of esophagus atresia, just as the case in our patient. Type III and IV clefts can be associated with other congenital anomalies including esophageal atresia, microgastria, and congenital heart disease as part of the VACTERL association. The surgeries of these patients can be more difficult, complicated, and are associated with a high risk of mortality [4–6]. Combination of lateral thoraco-cervical approach and anterior cervicothoracic approach is recommended. Lipshutz et al. have chosen to use an anterior midline transtracheal exposure to avoid recurrent laryngeal nerve injury and possible devascularization of the trachea and esophagus [3]. They believe this approach is safer than the lateral approach, although it is technically more demanding, and it adds a third suture line. The anterior approach is safe and technically feasible for long clefts. Intraoperative airway control with an open trachea is performed with intubation across the operative field, similar to the techniques used during the repair of tracheal stenosis. A tracheoesophageal fistula can occur despite meticulous attempts to avoid overlapping suture lines, most likely because of the tracheostomy tube. Thus, one may consider endotracheal intubation only postoperatively, although prolonged intubation may endanger the reconstructed larynx. Prolonged postoperative mechanical ventilation may be necessary because of tracheomalacia and bronchomalacia, not secondary to an anatomic abnormality or vocal cord paresis.

Tracheostomy is contraindicated in anterior approach since it erodes the posterior wall [3]. In the series of Mathur with nine patients, it has been detected that there is a direct relationship between the length of the cleft and survival. While four patients with a LTEC that ended above the carina survived after the repair, five cases where the cleft extended to the carina were lost [4]. We operated on second patient with an anterior approach due to reasons cited above. However, we had to extend the sternal incision so as to see the 1.5 cm-neck incision, trachea, and the carina better on the manubrium sterni that started from the level of the larynx in the shape of a T, which was recommended in surgery. Moreover, it was difficult to separate both the cleft and the esophagus since the cleft was extending on the level of the carina towards the right bronchus when the trachea was separated into two. In the meantime, the patient could not be well oxygenated, although an effective intubation was performed with right and left endotracheal intubation intraoperatively. Repair with cardiopulmonary by-pass or extracorporeal mebrane oxygenation(ECMO) that are recommended during the surgery of these kinds of patients was not preferred owing to the complications the patient would go through along with technical difficulties and the littleness of the patient [1, 2, 6, 7]. Even though the two incisions made to the cleft anteriorly and laterally during anterior approach are sufficient for the development of new trachea and esophagus, separation of the trachea and esophagus are difficult in cases like ours where the cleft extends to the tracheal carina. Therefore, in terms of both airway and seeing the esophagus and trachea separation better laterally, it could have been better if the trachea had been approached with a lateral thorcocervical incision rather than an anterior one. According to our knowledge in our country, our second patient was the first successful surgery done for LTEC type IV, but the patient was lost on postoperative day tw. However, patients with long clefts are usually lost as shown in the literature.

## Conclusion

Our first choice of approach was anterior cervicothoracic in the repair of type IV LTEC. This approach enables good exposure and less complications in cleft repair. Yet, lateral thoraco-cervical approach could have been better in the repair of clefts that extend to the carina as r.
